# Enabling Agile Clinical and Translational Data Warehousing: Platform Development and Evaluation

**DOI:** 10.2196/15918

**Published:** 2020-07-21

**Authors:** Helmut Spengler, Claudia Lang, Tanmaya Mahapatra, Ingrid Gatz, Klaus A Kuhn, Fabian Prasser

**Affiliations:** 1 Institute of Medical Informatics, Statistics and Epidemiology University Medical Center rechts der Isar, School of Medicine Technical University of Munich Munich Germany; 2 Charité - Universitätsmedizin Berlin Berlin Germany; 3 Berlin Institute of Health Berlin Germany

**Keywords:** cohort selection, hypothesis generation, data warehouse, translational research, hosting, Docker, extract-transform-load, i2b2, tranSMART

## Abstract

**Background:**

Modern data-driven medical research provides new insights into the development and course of diseases and enables novel methods of clinical decision support. Clinical and translational data warehouses, such as Informatics for Integrating Biology and the Bedside (i2b2) and tranSMART, are important infrastructure components that provide users with unified access to the large heterogeneous data sets needed to realize this and support use cases such as cohort selection, hypothesis generation, and ad hoc data analysis.

**Objective:**

Often, different warehousing platforms are needed to support different use cases and different types of data. Moreover, to achieve an optimal data representation within the target systems, specific domain knowledge is needed when designing data-loading processes. Consequently, informaticians need to work closely with clinicians and researchers in short iterations. This is a challenging task as installing and maintaining warehousing platforms can be complex and time consuming. Furthermore, data loading typically requires significant effort in terms of data preprocessing, cleansing, and restructuring. The platform described in this study aims to address these challenges.

**Methods:**

We formulated system requirements to achieve agility in terms of platform management and data loading. The derived system architecture includes a cloud infrastructure with unified management interfaces for multiple warehouse platforms and a data-loading pipeline with a declarative configuration paradigm and meta-loading approach. The latter compiles data and configuration files into forms required by existing loading tools, thereby automating a wide range of data restructuring and cleansing tasks. We demonstrated the fulfillment of the requirements and the originality of our approach by an experimental evaluation and a comparison with previous work.

**Results:**

The platform supports both i2b2 and tranSMART with built-in security. Our experiments showed that the loading pipeline accepts input data that cannot be loaded with existing tools without preprocessing. Moreover, it lowered efforts significantly, reducing the size of configuration files required by factors of up to 22 for tranSMART and 1135 for i2b2. The time required to perform the compilation process was roughly equivalent to the time required for actual data loading. Comparison with other tools showed that our solution was the only tool fulfilling all requirements.

**Conclusions:**

Our platform significantly reduces the efforts required for managing clinical and translational warehouses and for loading data in various formats and structures, such as complex entity-attribute-value structures often found in laboratory data. Moreover, it facilitates the iterative refinement of data representations in the target platforms, as the required configuration files are very compact. The quantitative measurements presented are consistent with our experiences of significantly reduced efforts for building warehousing platforms in close cooperation with medical researchers. Both the cloud-based hosting infrastructure and the data-loading pipeline are available to the community as open source software with comprehensive documentation.

## Introduction

### Background

Digitalization of health care promises to enable personalized and predictive medicine [1]. On the basis of digital data that characterize patients and probands at comprehensive depth and breadth [2], modern methods of data analytics can be used to detect unknown relationships between biomedical parameters, discover new patterns, and enable decision support systems by using this knowledge to infer or predict parameters, for example, diagnoses or outcomes [3,4]. A *learning health system* [5], which makes health care data available for secondary research purposes, is an important building block of this development. By comprehensive data integration within and across sites, a massive change in clinical and research processes is envisioned, which will accelerate translation and lead to measurable benefits for patients [6]. In this study, we focus on the integration of structured, that is, typically tabular, clinical and research data.

Multiple technical challenges must be addressed to provide the large, high-quality data sets needed for such purposes. Data from distributed and heterogeneous sources must be integrated at the technical, structural, and semantic levels [7]. To this end, a 3-step extraction-transformation-loading (ETL) process is often implemented:

1. Data from research and health care systems are transferred into a staging area in the form of nearly exact copies of data extracted from the source systems [8].
2. Within the staging area, the structure, syntax, and semantics of these data extracts are then normalized into a common data model (CDM) using standard terminologies. These common data representations typically implement a specific database schema, which efficiently and effectively supports complex analytical query processing.
3. Finally, the data are loaded into the target system.

Important examples include clinical and translational data warehousing platforms, such as Informatics for Integrating Biology and the Bedside (i2b2) [9], tranSMART [10], and the Observational Medical Outcomes Partnership (OMOP) CDM [11]; federated and distributed solutions, such as the Shared Health Research Information Network [12]; and the tools provided by Observational Health Data Sciences and Informatics (OHDSI) [11], which can be deployed on top of these analytical databases.

These existing biomedical data analytics platforms offer a wide range of functionalities and integrate different software solutions for data storage, workflow orchestration, and data analysis using multi-tier architectures. As a result of this complexity, considerable technical expertise is required to set them up in a secure manner. These challenges increase even further when organizations run several data-driven research projects and hence need to set up, configure, and maintain multiple warehouse instances. Moreover, ensuring that input data are represented in the analytics platforms in a sound structure with reasonable semantics requires significant medical expertise. It is well known that bridging the interdisciplinary gap between these two worlds requires iterative development processes, in which different solutions are evaluated in short feedback cycles [13]. As we will show later, existing data-loading tools for the aforementioned platforms, however, typically require complex configuration files and input data that adhere to specific formats and structures. Consequently, substantial data restructuring and cleansing is required before data loading can be started and initial feedback can be collected.

**Figure 1:**
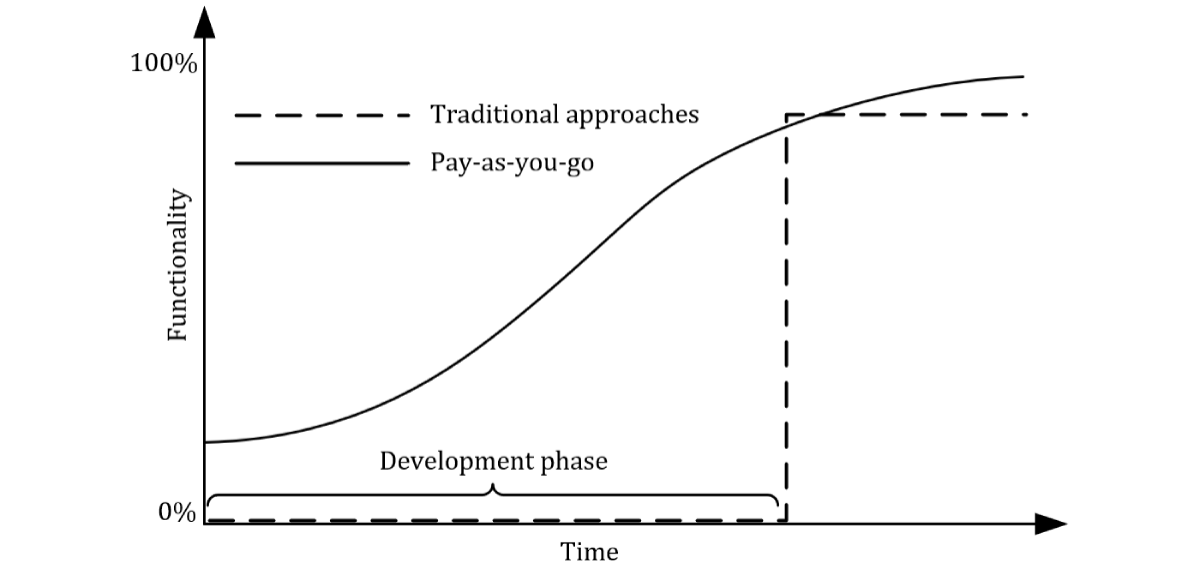
Schematic comparison of traditional approaches to data integration and the pay-as-you-go approach.

In an ideal world, upfront efforts for project-specific technical setup, data cleansing, and data structuring can be avoided, and development starts rapidly, while repeated discussions with clinicians and medical researchers are carried out in parallel [14]. Technical solutions that facilitate this approach have been called *dataspace management systems* [15]. The key idea is to implement a *pay-as-you-go* approach to data integration. A comparison with traditional approaches is presented in Figure 1. It illustrates how the traditional approaches are characterized by an initial development phase in which the data are being integrated on a syntactic, structural, and semantic level, and no service is provided to the users. In contrast, the pay-as-you-go approach provides some initial functionality from the beginning, which is then incrementally extended to better meet the requirements [15,16]. This means that the associated development process can be carried out in an agile manner, involving close cooperation and short feedback cycles with end users. This comes with multiple benefits for the parties involved: clinicians or medical researchers are provided with initial functionalities much more quickly, and feedback can be provided to the development team more often. This is particularly important for data loading because it has been estimated that the development of ETL processes accounts for up to 70% of the total effort required to set up data warehouses [7,17]. For both end users and developers, this can also lead to the reduction of duplicate and redundant work, thus significantly reducing the efforts required. The approach is related to agile methods of software engineering, in which software evolves through continuous collaboration between developers and users. It is well known that this can also help to better bridge the interdisciplinary gaps [18].

### Objectives

The aim of this study was to implement a platform that enables the deployment and customization of well-known clinical and translational data warehousing solutions in close cooperation with end users in an agile approach. Our solution consists of 2 parts with the following unique features:

1. A cloud-based warehouse management infrastructure, which supports the installation and maintenance of i2b2 and tranSMART in an integrated manner by providing a common set of commands; implements security-by-default features, including transport layer encryption, host-based access control, and password management; and is based on verifiable and authenticatable software to enable installations within high-security perimeters of hospital information technology (IT) environments.
2. A flexible data-loading pipeline, which supports loading data into both i2b2 and tranSMART; is able to process heterogeneous data with different degrees of structure and cleanliness; and performs automated data cleansing and preprocessing, including automatic detection of the syntax and format of input data, and has the ability to handle different encodings as well as missing and duplicate data.

The complete software stack is available to the community as open source software [19,20]. In this study, we provide readers with an overview of the most important system requirements and design decisions. To demonstrate that our solution enables an agile approach to be implemented in a professional context, we present the results of a structured comparison with existing management infrastructures and data-loading pipelines as well as an experimental evaluation of data-loading processes. Our results show that our management infrastructure is the only publicly available open source implementation that supports all the abovementioned features, which is essential for secure deployments in professional IT environments. Moreover, the experimental evaluation showed that no other open source data-loading pipeline was able to process 3 different benchmark data sets, including structured research data, complex longitudinal clinical data, and highly structured billing data, in their raw form. The experiments also showed that our solution is feasible from a computational perspective. We believe that the software presented in this study can be an important tool to support medical informaticians with realizing data warehousing projects and that the methods implemented can provide system developers with novel ideas for the development of future platforms.

## Methods

### Selection of Target Systems

Clinical and translational data warehouses provide users with efficient analytical access to integrated data sets [21,22]. As an initial step, we decided to utilize an infrastructure supporting i2b2 and tranSMART as both of these have a broad installed base and strong community support. For example, the integrated solution of Hôpital Européen Georges-Pompidou [23] uses i2b2 and tranSMART, integrating data from electronic patient records, including aggregated, anonymized, and *deanonymized* patient data. The tranSMART platform [10] is based on the i2b2 framework, and its suitability for data from clinical studies has already been demonstrated in various projects [24]. In combination, they can be used to support a wide range of use cases.

The i2b2 platform is very well suited for representing longitudinal and often semistructured clinical data, and it supports complex features such as temporal queries against time series data [9]. TranSMART was built over the i2b2 data model to provide improved support for high-dimensional data. The system is well suited for integrating structured research data as well as high-throughput data, and it provides comprehensive support for ad hoc graphical data analysis and cohort comparison [10]. TranSMART offers built-in support for various types of omics data, such as protein and gene expression arrays, single-nucleotide polymorphism data, and certain types of genomic variants. With the recent merger of the i2b2 Foundation and the tranSMART Foundation, a process has been started to unify both platforms. Until a combined solution becomes available, installations of both systems are needed to support different use cases and to handle different types of data.

The 2 systems offer web-based graphical user interfaces. TranSMART employs a classical three-tier information system architecture, whereas i2b2 consists of an extendable framework consisting of several *cells*. Both platforms can be installed on top of different database management systems. As we focus on open source software, we decided to use PostgreSQL, an open source relational database management system.

### Cloud Infrastructure for Managing i2b2 and tranSMART

#### Rationale and Requirements

Both i2b2 and tranSMART offer a wide range of functionalities, and they are based on a software architecture that integrates components for data storage, workflow orchestration, and data analysis. Consequently, installation, configuration, and maintenance procedures are complex and require solid technical expertise. Concurrently, documentation is often lacking. As an example, the number of tranSMART software dependencies is very large, which regularly leads to some dependencies not being up to date or having become incompatible with the underlying (operating) system infrastructure, requiring manual changes to installation scripts. In contrast, the i2b2 installation process is fairly robust, well documented, and up to date [25]. However, it can be quite challenging to debug configuration errors of i2b2 owing to its highly modular architecture, which involves exchange of complex data via web services. These challenges increase significantly when a larger number of instances need to be set up, configured, and maintained. Furthermore, when deploying such systems in production environments, additional aspects such as transport encryption and password management need to be considered. These and further functionalities are not supported by existing cloud-based deployment solutions for i2b2 and tranSMART, such as the Integrated Data Repository Toolkit (IDRT) [26], i2b2 Quickstart [27], or the prebuilt images available on Docker Hub [28] (see the *Discussion* section for an in-depth comparison).

We, therefore, decided to employ clean virtual containers, ideally together with associated maintenance scripts to quickly boot up, configure, and shut down instances of i2b2 and tranSMART in a uniform manner. The most important requirements were as follows:

1. *Robust installation of a trusted runtime environment:* The solution developed shall streamline the complex installation process of tranSMART and enable rapid instantiation of new instances of tranSMART and i2b2.
2. *Unified installation and maintenance:* The solution shall provide a façade encapsulating important configuration options and make the effective management of multiple instances of i2b2 and tranSMART straightforward by providing easy-to-use common commands for both platforms.
3. *Built-in security:* The solution shall significantly improve the security of i2b2 and tranSMART by enabling transport encryption and host-based access control by default as well as by automatically setting nontrivial passwords.

#### Technical Design

The cloud infrastructure has been designed to run on a physical or virtual machine with a standard Linux operating system. In this system, Docker needs to be installed as a virtualization platform that enables the provisioning of software in deployment units called containers. Each container encapsulates a complete software stack together with all required dependencies, such as libraries and configuration files. Docker employs OS-level virtualization, which means that in contrast to full virtualization, where each virtual machine contains and runs its own operation system, Docker containers can share one single operating system instance and are thus more lightweight than virtual machines. Although containers are isolated from each other, they can be enabled to communicate through definable channels (eg, Transmission Control Protocol ports). Containers can quickly be instantiated and customized via runtime parameters in this process.

We chose Docker for the following reasons: (1) it enables describing and documenting installation processes in a machine and human-readable format, thus fulfilling our requirement for robust installation and quick instantiation; (2) it allows customizing running containers by means of runtime parameters (eg, access permissions, passwords, and instance names), thus fulfilling our requirement to provide uniform configuration and maintenance scripts for both platforms; (3) its efficient use of resources allows rapid booting up and shutting down instances; and (4) it integrates well with common software development infrastructures, such as GitLab.

As a gateway component to provide transport encryption, host-based access control, and data routing for the particular warehouse instances, we decided to include the Apache HTTP Server into the host environment utilizing its proxy and virtual host modules.

##### Meeting Requirement 1: Robust Installation of a Trusted Runtime Environment

The solution can be used to host an arbitrary number of i2b2 and tranSMART instances. Each host system includes the following containers per instance: (1) a database server for i2b2, (2) an application server for i2b2, (3) a web server for i2b2, and (4) a complete tranSMART software stack. It can be accessed via specific URLs. The subdomain in this URL denotes the warehouse instance, for example, *dwh01* or *dwh02*. Each subdomain is represented by a dedicated Apache virtual host and provides one instance of i2b2 and one instance of tranSMART. As an example, the URL-pattern *[http|https]://dwh02.example.org/i2b2/* denotes the web front-end of i2b2 instance *02*, which is exposed by the Apache virtual host *dwh02.example.org*.

Both tranSMART and i2b2 expose specific ports to provide specific services. These include their web front-ends and various web services. To avoid port clashes when running multiple warehouse instances and their respective containers, the ports used by each container are mapped to corresponding ports on the host system using specific offsets such that a certain set of ports uniquely identifies each service of each container.

**Figure 2:**
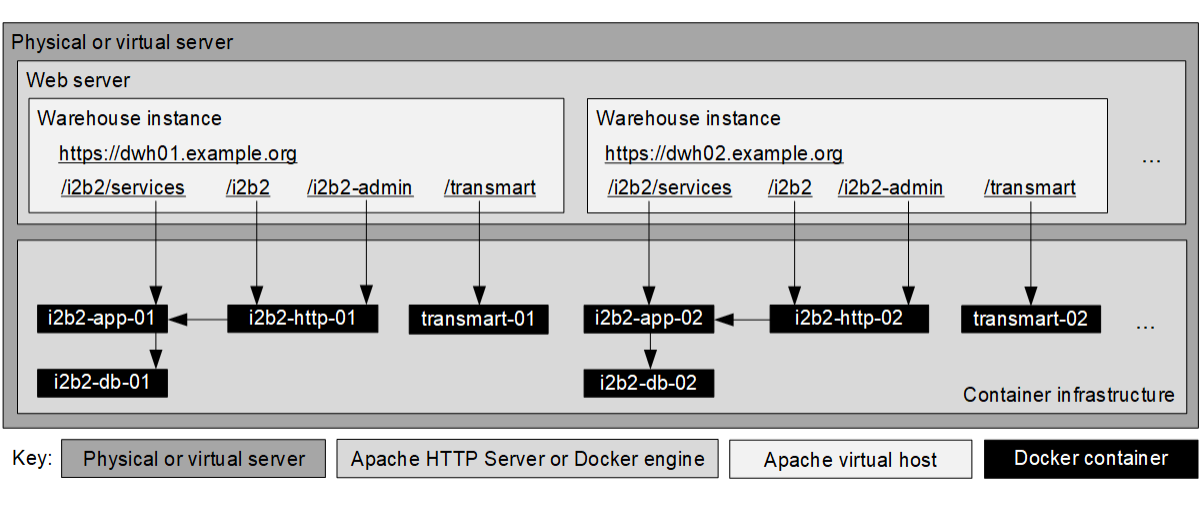
Schematic overview of the components for the provisioning of multiple warehouse instances and their interaction.

Figure 2 illustrates the components used by the environment and their interactions. The actual instances of i2b2 and tranSMART are implemented as (stacks of) Docker containers (black boxes). Access to these containers is relayed by an Apache web server, which acts as a gateway. Each warehouse instance is represented by a virtual host of the gateway and is identified by the first part of the hostname contained in the URL of the request. Detailed installation instructions along with well-documented configuration files are available on the web [19].

##### Meeting Requirement 2: Unified Installation and Maintenance

To support unified management for instances of both types of systems, we have developed 2 configuration scripts that can be parameterized. Target instances are identified by their type and consecutive numbers (eg, i2b2-04). The first script can be used to set up new warehouse instances and to reset existing instances. It does so by creating configurations for Apache’s proxy and virtual host modules and environment files for the Docker compose scripts. If needed, the resulting files can be edited by the administrator (eg, to replace randomly generated passwords) before the new instances are created. The second script can be used for starting, stopping, and deleting warehouse instances as well as associated disk volumes. It has been implemented as a wrapper for Docker compose commands that access the environment variables defined in the associated environment files.

##### Meeting Requirement 3: Built-In Security

The setup process implements several crucial security measures, including transport layer encryption, server authentication, restricted access paths, and nontrivial default passwords.

Access to the services running on each server is only permitted indirectly via the Apache HTTP Server, which acts as a central gateway. This component takes care of the transport encryption and server authentication mentioned above as well as address-based access control. The only service that can be reached without having to pass the gateway is the database system to enable efficient data loading. Here, access control is implemented at the database level. Permission to access the database has to be granted explicitly, which includes the declaration of address ranges with specific access rights. To simplify the Transport Layer Security configuration, we make use of the *subject alternative name* extension to the X.509 server certificates [29], which our platform uses for authenticating the data warehouses and for transport layer encryption. Embedded plain text secrets and the fact that the source and content of many images cannot be verified have been identified as major risks for system components based on container technologies [30]. This impedes the use of prebuilt images in high-security IT environments. Our infrastructure does not suffer from these shortcomings as we employ Docker Content Trust [31] to verify the authenticity of all base images used. As the current images for i2b2 and tranSMART do not support this authentication mechanism, we decided to build our own images based on authenticated sources (by verifying Pretty Good Privacy signatures of binaries used and/or building them from source). Secure default passwords are automatically created via a random password generator [32] with a default length of 10 characters and injected into the containers at runtime.

### Generic and Agile Data-Loading Pipeline for i2b2 and tranSMART

#### Rationale and Requirements

Populating i2b2 and tranSMART with data is cumbersome and requires significant expertise regarding the underlying database schema and how both systems use it. For this reason, several tools have been developed to simplify this process, including tranSMART-ETL [33], tMDataLoader [34], transmart-batch [35], Integrated Curation Environment (ICE) [36], IDRT [26], transmart-copy [37], and TranSMART data curation toolkit (tmtk) [38]. However, none of these tools fulfill the requirements needed to implement agility (see the *Discussion* section for an in-depth comparison).

First, all available data loaders except transmart-batch are strongly tied to 1 of the 2 target systems. As both are often needed in parallel, this introduces additional preprocessing and configuration efforts. The main reason is that loaders for different systems make different assumptions about the degree of structure and cleanliness of import data. In addition, different loaders use different configuration mechanisms. Moreover, existing tools follow imperative configuration paradigms, where it must be specified how the loading process should be executed, making this process complex and requiring substantial technical expertise as well as domain knowledge. Finally, to support agile and fast loading, tools should be able to automatically handle heterogeneity and errors in input data, such as differences in data encoding and syntax as well as missing and duplicate data. To address these challenges, we needed a data-loading pipeline fulfilling the following requirements:

1. *Platform independence:* The data-loading pipeline shall be designed independent of a specific target system, enabling the loading of data into both i2b2 and tranSMART with the same pipeline using the same configuration files.
2. *Support for different types of data:* The pipeline shall support heterogeneous data with different degrees of structure and cleanliness, such as structured research data, complex longitudinal clinical data, and highly structured billing data, without requiring complex preprocessing or configuration efforts.
3. *Automated data cleansing and preprocessing:* The pipeline shall automatically detect the syntax and format of input data and handle different encodings as well as missing and duplicate data. This significantly reduces efforts and improves agility when providing warehousing solutions.

#### Technical Design

The most important design decision made to fulfill the requirements listed above was to center the tool around a declarative and model-driven way of configuring the import process. The basic idea was to enable users to match data to an entity-relationship (ER) model that describes the desired target representation of the data. The tool then automatically determines how the input data must be interpreted, transformed, and loaded to reflect this model in the target database. This includes the automatic creation of the ontologies required by i2b2 based on this model. This is in stark contrast to the imperative configuration paradigm found in most ETL tools for i2b2 and tranSMART and significantly reduces the complexity of configuration files and hence efforts (see the *Results* and *Discussion* sections). Moreover, the approach enables our tool to automatically perform a wide range of data transformation and cleansing tasks, thus fulfilling our requirements to support different types of data and automate data cleansing. To fulfill the requirement of platform independence, our tool acts as a *compiler* for configuration files to be used for different ETL tools for i2b2 and tranSMART.

The data-loading tool has been developed in Java using the Spring Batch framework for robust, maintainable, and extensible orchestration of the individual steps of the ETL process; the Univocity parser for reading and writing comma-separated values (CSV) files; and juniversalchardet, a Java port of Mozilla’s library, for the automatic detection of file encodings. Access to the target relational database systems has been implemented using Java Database Connectivity.

##### Meeting Requirement 1: Platform Independence

As some powerful loading tools for the different target platforms have already been developed, we decided to implement a meta-loading process consisting of 2 phases: the first is the *staging* phase, in which data are transformed into an intermediate staging representation and configuration files are compiled into the target configuration language for the respective loading tool, which we term *back-end* loader in the context of our meta-loading process. We refer to the transformed data and the configuration files created in this phase as s*taging files*. The second is the *loading* phase, in which the staging files are used to execute the respective back-end loader for the chosen target platform.

**Figure 3:**
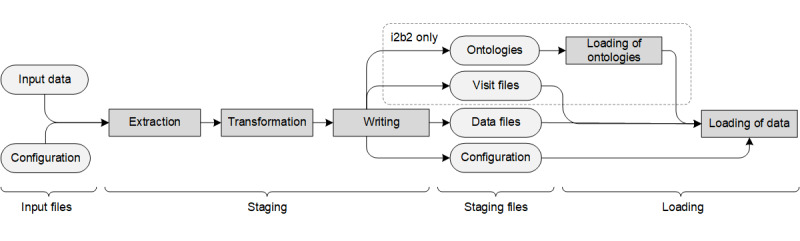
Overview of data staging and loading with the tool developed. i2b2: Informatics for Integrating Biology and the Bedside.

Figure 3 illustrates a typical staging and loading process. The *staging phase* is divided into 3 subphases: data extraction, data transformation, and data writing. In the data extraction subphase, our tool reads the declarative configuration, which describes the structure of data to be represented in the target system. On the basis of this configuration, it reads and parses the input data. Details are presented in the 2 subsequent sections. In the data transformation subphase, different data cleansing steps are performed, which are also be presented in the 2 subsequent sections. The last subphase involves writing the transformed data into intermediate files, which are consumed by the back-end data loaders in the loading phase. In the case of i2b2, visit data are written separately. This is followed by writing the associated configuration files, describing how the staging data are to be loaded. In the case of i2b2, this (pre-)final step is concluded by writing data describing the underlying ontologies into separate files. In the *loading phase*, the actual data loading is performed by executing the user-defined back-end loaders. If i2b2 has been selected as the target system, this step is preceded by loading the ontology trees into the target system. Currently, our tool supports the following 2 back-ends for data loading:

- *tMDataLoader*, which has been implemented in Groovy and in stored procedures of the underlying database system to automate data loading for tranSMART [34]. The tool relies on a specific directory structure, containing the data sets and configurations, thus following the convention over configuration approach. It supports the full spectrum of features provided by tranSMART, including the annotation of selected values with timestamps.
- *transmart-batch*, which is implemented in Groovy using Spring Batch and which has been designed to support both tranSMART and i2b2. It requires a specific set of files to be provided about subjects and visits as well as further files containing the actual payload data. It supports fewer features of tranSMART than tMDataLoader and requires significant data cleansing to be performed upfront to provide data in the syntax and structure required.

##### Meeting Requirement 2: Support for Different Types of Data

As mentioned before, the configuration is performed using a *declarative* approach [39]. This means that users do not need to specify how data should be loaded, but instead map an ER model to the data files to describe the relationship between input and output data. Consequently, the tool can perform a wide range of data transformations automatically without prior normalization, including the automatic creation of the target ontology. Although users are less flexible in defining how data should be represented in the target system, a decent representation can typically be achieved for almost all of the data items, as we will show later, with just a fraction of the effort required to use a more versatile loader. If needed, users can still modify and fine-tune the intermediate staging files to achieve an optimal representation.

The tool developed was designed in such a way that the maximum degree of the work that needs to be done for successful loading is automated. There are just a few assumptions that are made about input data: (1) data must be tabular, as this is in our experience the most typical format in which clinical and research data can be provided; (2) every line within a file must contain data for a specific patient, visit, or encounter; (3) patients, visits, or encounters must be identified by (composite) keys or timestamps; (4) one file must contain information about the patients or probands—a file describing visits or encounters is optional; and (5) entities may be related to patients, visits, or encounters. Providing information on time points is optional but recommended.

**Figure 4:**
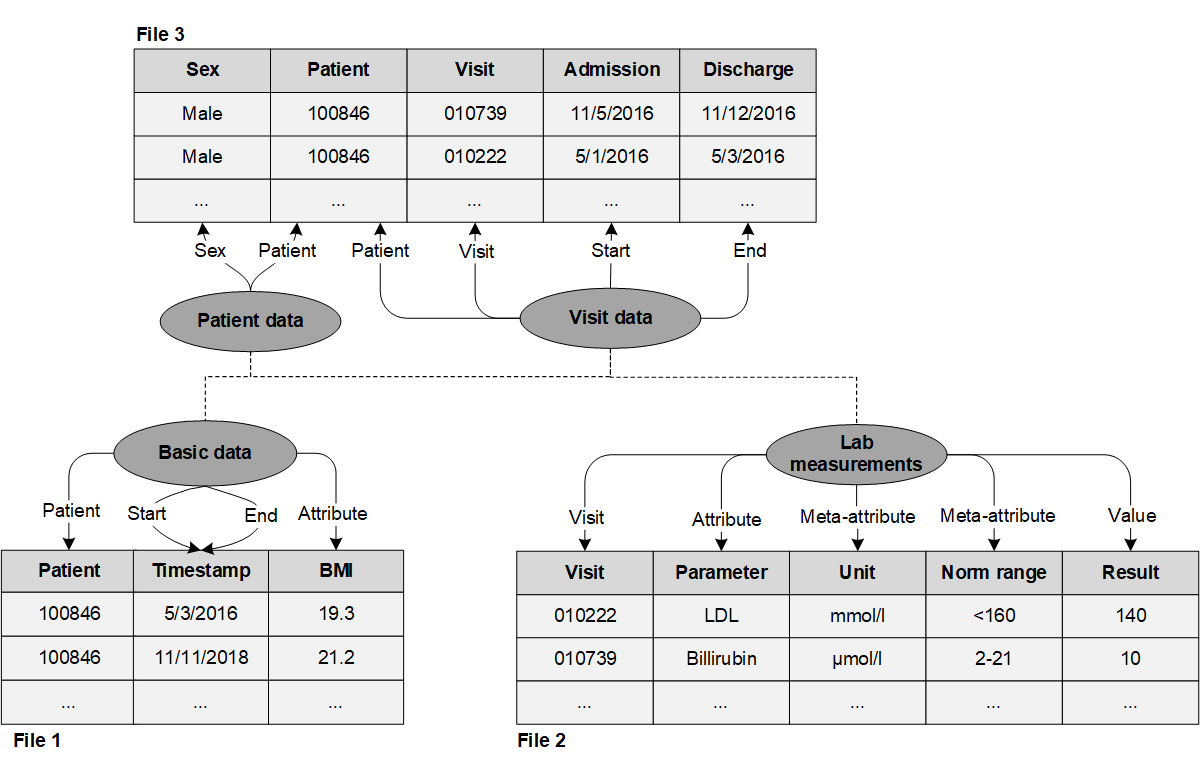
Simplified example of an annotation of input files with entities, attributes and relationships. LDL: low density lipoprotein.

Figure 4 provides an example of how the tool is configured. As can be seen, users are able to specify *entities* that are related to a certain patient or visit and that have *attributes*. Attributes can be mapped to specific columns in the input files. Attributes can be annotated with *meta-attributes*, which are attributes that further specify a specific value for an attribute of a specific entity. In i2b2, these are mapped to *modifiers*. Although there is no direct support for meta-attributes in tranSMART, they can in some cases be represented by creating multiple variants of an attribute that encodes the values of the associated meta-attributes. In addition, there are specific attributes for specifying timestamps and patient or visit identifiers.

The figure also shows an example of how data stored in an *entity-attribute-value* (EAV) model can automatically be denormalized. The EAV model is often used in data collection systems when a large number of different observations are recorded but only a few of them typically apply to a specific patient or proband (eg, lab values). To support this, an additional property *value* is introduced, which can be used to specify how data in EAV form should be denormalized. In the example, one entity will be created in the target systems for each instance of the column *Parameter* having the value from the column *Result* and being annotated with meta-attributes *Unit* and *Norm range*. This is implemented by parsing the input files and populating the configuration with automatically generated parameters for each EAV-encoded data item.

By specifying basic patient, visit, and observational data, the specified EAV entities, the patient data, the observations, an internal model of the ontology, and optionally the associated visits are automatically created. Furthermore, by mapping patients to visits and by relating entities to visits or patients, implicit relationships between the different types of data are constructed. These will also be reflected within the target systems.

##### Meeting Requirement 3: Automated Data Cleansing and Preprocessing

There are multiple additional features that have been added to the tool based on our experiences with loading a wide range of real-world data sets, which help enforce the syntactic and structural integrity of the input data and which are particularly important due to the heterogeneity of the data sources with respect to these parameters. Important examples include the automated detection of charsets and syntax of input data as well as the automated detection of data types of variables. Features that help enforce semantic integrity include the detection and handling of duplicate data, inconsistent timestamps, and missing values. Finally, support for data filtering and methods for handling uncertainty in timestamps are provided. On a technical level, these tasks are executed as part of either the data loading or the data transformation subphase.

### Experimental Design

We evaluated our solution by performing an experimental evaluation of our data-loading approach using different real-world data sets. In the experimental evaluation, we focused on 3 different aspects:

1. *Flexibility:* To demonstrate that our loading tool is able to perform automated data cleansing and restructuring, we used it to load three different types of data sets with varying degrees of structure and cleanliness. Moreover, we also tried to load these data sets using existing data-loading tools to demonstrate that they are not able to process them without prior data cleansing.
2. *Reduced efforts:* To demonstrate that the declarative configuration paradigm of our loading tool significantly reduces the effort required, we compared the number of lines in the configuration files for our tool with the number of lines of the configuration files generated for and needed by existing data-loading tools.
3. *Scalability:* To demonstrate that our approach is computationally feasible, we compared the time needed for automated data cleansing and preprocessing with the time required for actual data loading.

In the experiments, we used real-world data sets from 3 different previous projects: (1) a research data set including *microbiome profiles*, (2) clinical data on *multiple sclerosis*, and (3) *billing data*.

The microbiome profile data set was collected in a study context by our internal medicine department in 2019 and included general information about the probands, lifestyle information obtained through questionnaires, and microbiome profiles (species identified by 16S rRNA gene sequencing) generated from sampled stool, feces, and esophagus tissue. The multiple sclerosis data set was collected by our neurology department since 2010 in the health care context and consisted of longitudinal clinical data, including diagnoses, procedures, clinical scores, medication, lab values, references to biosamples, and metadata of imaging tests. The billing data set consisted of discharge data collected in our hospital in the years 2015-2017 containing demographics and visit data including ventilation time, diagnoses, and procedures. Further details on the projects and use cases supported by these data sets are presented in the *Discussion* section.

For loading data into i2b2, we used the transmart-batch backend, and for loading data into tranSMART, we used the tMDataLoader backend of the pipeline. The experiments were performed with the warehouse instances hosted on a server with Intel Xeon central processing units (CPUs) running at 2.4 GHz with 80 cores, along with 512 GB RAM and 16 TB hard-drives using kernel-based virtual machines provided by Quick EMUlator 2.5.0 running on Ubuntu 18.04. The ETL processes were executed on a desktop machine equipped with a quad-core 3.2 GHz Intel Core i5 CPU running a 64-bit Windows NT kernel, with a 32-bit Java Virtual Machine (1.8.0_202_x86), and with the data input files located on the local file system.

## Results

### Experiment 1: Flexibility of the Loading Process

In this section, we present results on the flexibility of the loading process for our evaluation data sets and both i2b2 and tranSMART as target systems. The basic properties of the data sets and their representations in the target systems are shown in Table 1.

The microbiome data set originates from a study context and is highly structured. For this reason, and as can be seen in Table 1, i2b2 and tranSMART were both fully able to represent the data set as is. The multiple sclerosis data set, in contrast, was collected in the health care context and consisted of longitudinal clinical data with less structure and a multitude of detailed measurements, such as laboratory values. As can be seen in Table 1, tranSMART could only capture parts of these data (fewer facts by a factor of 6 compared with i2b2) because of missing support for complex time series data and meta-attributes. The billing data set was also highly structured and contained dates of admission and discharge as well as coded diagnoses and procedures. In general, these data could be represented well in i2b2 as well as tranSMART, but the latter system was not able to capture meta-attributes, for example, of diagnoses, resulting in some loss of information.

We emphasize that loading into the different target systems was achieved using the same configuration files. We conclude that our tool provides a high degree of flexibility but that the different target systems are not able to capture all aspects of input data. In general, i2b2 is more suited for representing longitudinal clinical data, and tranSMART is better suited for analyzing highly structured research data.

We further emphasize that our loading pipeline was the only tool with which we were able to load all the data sets described in their raw form without prior transformations or preprocessing. In the remainder of this section, we will briefly cover the issues encountered when using existing open source loading software. We present a detailed comparison with our approach in the *Discussion* section.

When loading the data sets into i2b2, we encountered the following issues: transmart-batch for i2b2 requires the extraction and loading of concept trees into i2b2 before the import of the actual facts. This process is not supported by the tool, and import files also need to be annotated with codes associated with the ontology nodes in the database in an additional preprocessing step. The loading pipeline of IDRT is no longer maintained (over 2.5 years old) and is not compatible with i2b2 1.7.09c and higher, resulting in various errors during data loading. When loading the data sets into tranSMART, we noticed the following problems: tMDataLoader, tmtk, transmart-batch, and ICE could not load the clinical data set where multiple values were provided for the same variable and subject in the same visit. Furthermore, values are required to conform to predefined formats (eg, “yyyy-mm-dd hh:mm:ss” for dates), requiring preprocessing. Transmart-copy could not load any of the data sets used in our experiments without significant preprocessing at the structural and syntactical level, as it required input data to precisely conform to the target schema. TranSMART-ETL could also not load the clinical data set as it was not able to handle missing values. Moreover, it required specific column separators and number formats to be used, requiring input files to be preprocessed accordingly.

**Table 1:** Overview of the properties of the data sets used in the projects.

Data set	Microbiome profiles	Multiple sclerosis	Billing data
Number of input files	15	19	11
Size of input files in MB	1	497	252
Patients	~50	~7000	~100,000
Visits	~100	~40,000	~300,000
Facts in i2b2^a^	~90,000	~4,600,000	~6,200,000
Facts in tranSMART	~90,000	~750,000	~3,800,000

^a^i2b2: Informatics for Integrating Biology and the Bedside.

### Experiment 2: Reduction of Efforts

In this section, we present the results of the reduction of efforts that can be achieved by using our loading tool. We captured this aspect by analyzing the size of files used for actual data loading, which are shown in Table 2. It shows the complexity of configuration files required for data loading with our tool compared with the complexity of the configuration files generated for the backing data loaders. As can be seen, the tool presented in this study generated a large number of files for the different specified entities. Moreover, as a result of the automated denormalization of EAV data and the automated detection of data types, configuring data loading with our tool required significantly fewer lines of configuration parameters than what would have been required using transmart-batch or tMDataLoader. The configuration files for tranSMART for the multiple sclerosis and the billing data sets were much smaller than the corresponding files for i2b2, as they did not include specifications for meta-attributes.

For the microbiome data set, configuration files for our tool were smaller by factors of between 17.7 (i2b2) and 22.1 (tranSMART). For the multiple sclerosis data set, configuration files for our tool were smaller by factors of between 3.9 (tranSMART) and 216.1 (i2b2). For the billing data set, configuration files where smaller by factors of between 1.2 (tranSMART) and 1135.0 (i2b2). We note that the sizes were (roughly) equal only for the billing data set and tranSMART, which is because this data set is highly structured and because this type of data is well supported by tranSMART. We conclude that our tool can significantly reduce the efforts required for configuring the loading process.

**Table 2:** Comparison of input required for data loading.

Data set	Microbiome profiles	Multiple sclerosis	Billing data
LOC^a^ input	496	1090	83
LOC staging, i2b2^b^	8772	235,582	94,213
LOC staging, tranSMART	10,976	4272	99
Input files	15	19	11
Staging files, i2b2	2207	1034	31
Staging files, tranSMART	2194	854	18

^a^LOC: lines of configuration.

^b^i2b2: Informatics for Integrating Biology and the Bedside.

### Experiment 3: Scalability

In this section, we present the results on the scalability of our tools with respect to increasing volumes of data. The execution times measured in the experiments are provided in Table 3.

The table shows the time needed for staging and loading the data from the 3 evaluation data sets for i2b2 and tranSMART. As can be seen, the execution times scaled roughly linearly with the number of facts loaded into the target systems. Moreover, the relative time needed for data staging was the highest for the multiple sclerosis data set, which is also the data set with the highest complexity, thus requiring the most preprocessing.

**Figure 5:**
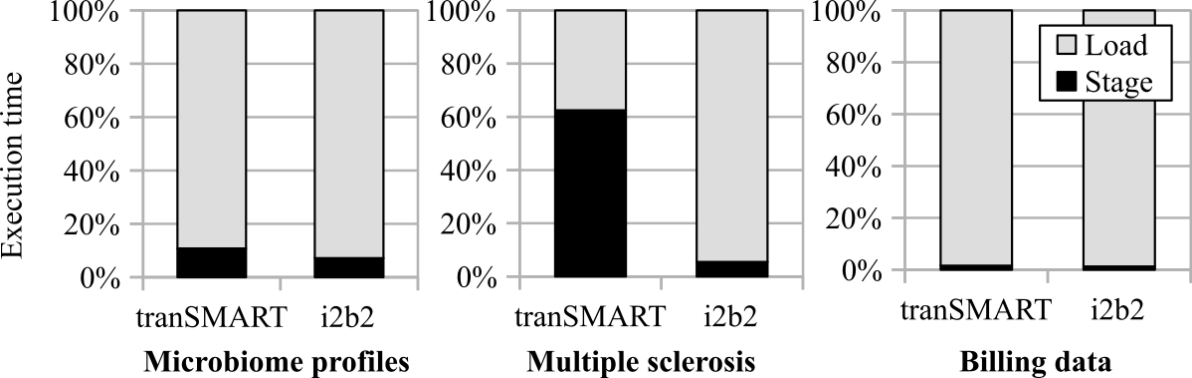
Percentage of loading and staging times regarding the complete process. i2b2: Informatics for Integrating Biology and the Bedside.

Figure 5 provides an overview of the relationship between the times needed for staging and loading. As can be seen, the (relative) staging times for tranSMART were generally higher than those for i2b2. This can be explained by the fact that more data normalization and restructuring were needed to be performed by the tool to ensure that the data could be loaded into the target system. In addition, more complicated procedures for duplicate detection were needed, as there is little support for the time axis in tranSMART. In summary, we conclude that our approach is scalable and can be used to process large data sets.

**Table 3:** Execution times of data-loading processes in seconds.

Data set		Microbiome profiles	Multiple sclerosis	Billing data
**tranSMART**				
	Staging time	13	687	91
	Loading time	109	413	5687
	Total time	122	1100	5778
**i2b2^a^**				
	Staging time	11	804	790
	Loading time	144	13,895	61,417
	Total time	155	14,699	62,208

^a^i2b2: Informatics for Integrating Biology and the Bedside.

## Discussion

### Principal Findings

We have presented a comprehensive cloud-based platform and a flexible data-loading pipeline to enable the agile provisioning of clinical and translational data warehousing solutions. We have presented an extensive experimental evaluation, dealing with different types of data and targeting platforms with different data analytics capabilities. The results of our analysis show that the presented platform significantly simplifies the management of the supported data warehousing solutions and enables quick loading of data in various representations. This enables the development of such platforms in close cooperation with users based on short feedback cycles. The cloud-based hosting infrastructure and the data-loading pipeline are available as open source software.

The infrastructure and tools presented in this study and the data sets used in our experimental evaluation have been used to support a variety of real-world projects. In particular, the infrastructure is being used to support a large clinical research center [40] that studies shifts in the composition and activity of the microbial ecosystem focusing on clinical endpoints that are associated with well-documented changes in the gut microbiome (inflammation and cancer). For this purpose, a platform is being set up to provide researchers with integrated access to different types of data generated within the consortium. Moreover, our platform is being used within the DIFUTURE (Data Integration for Future Medicine) project to improve data availability and accessibility through an integrated view on health care and research resources, such as biobanks [6]. An important example of one of the use cases of the project is the development of an infrastructure for personalized optimal treatment of multiple sclerosis combined with efforts to better understand the disease in general. Finally, the billing data set has been used in a nationwide cross-site analysis aiming at the reproduction of published comorbidity scores and the descriptive analysis and visualization of the distribution of comorbidity scores as well as the distribution of rare diseases in Germany [41].

### Comparison With Prior Work

#### Analytics Platforms

Currently, our solutions support i2b2 version 1.7.09c and tranSMART version 16.3. In future work, we plan to add support for further warehousing platforms and further versions to support further use cases. An important system of interest is i2b2-tranSMART, which is the result of an initiative to integrate tranSMART with the i2b2 cohort selection services and improved support for managing time series data [42]. In theory, this would obviate the need to support 2 different systems (i2b2 and tranSMART) with a similar technological basis. However, i2b2-tranSMART is still under active development and is not yet suitable for deployment in production environments. It is planned to release this software directly as a Docker container; therefore, we expect little effort to integrate it into the presented environment.

The OMOP CDM and OHDSI toolset also provide an interesting target platform [11]. OHDSI is an international collaborative initiative aimed at making clinical data accessible to analytics efforts, also in distributed settings, to generate actionable insights for improving health care. The OMOP CDM is a CDM for consistently representing health care data from diverse sources by making the relationships between different concepts explicit [11]. The OHDSI project provides a wide range of analytics front-ends, such as ACHILLES (Automated Characterization of Health Information at Large-scale Longitudinal Evidence Systems) or Atlas, an open source application developed as a part of OHDSI intended to provide a unified interface to patient level data and analytics. Both are aimed at end users and can be deployed over the OMOP CDM. Supporting OMOP/OHDSI within the described cloud-based hosting infrastructure will not be too complex. Implementing an agile loading process, however, will be challenging as the OMOP CDM requires a significant amount of data normalization and encoding with standard terminologies. Finally, cBioPortal would be an important additional system to support as it provides a platform for interactive exploration of multi-dimensional genomics data sets, intending to also support rapid, intuitive, and high-quality access to molecular data and clinical data [43]. A dockerized version for the presented cloud environment has already been implemented, but integrating the software with our data-loading pipeline requires more work.

#### Cloud-Based Infrastructures

Regarding cloud-based management infrastructures for clinical and translational data warehousing, most studies focus on i2b2 only. The *i2b2 Wizard*, which is part of the IDRT, as well as i2b2 Quickstart aims to simplify installation, setup, and administration of single i2b2 instances. There are also images available on Docker Hub. However, as neither the source code of these images is publicly available in full nor can their authenticity be verified (eg, using Docker Content Trust [DCT]), we could not use them as a base for further development because of security considerations. For tranSMART, a large number of images are available on Docker Hub. However, they have not been maintained for some time, contain artifacts with unclear provenance, or their documentation leaves out important aspects.

We compared these alternative solutions with our approach with respect to the following criteria:

1. *Supported target platforms* indicates whether a solution can be used for the current major version of i2b2 (ie, 1.7.x) and/or tranSMART (ie, 16.3).
2. *Container-based* denotes whether the solution is encapsulated using container virtualization, which significantly increases the ease and robustness of the installation procedures.
3. *Security by default* covers 3 subcriteria—whether *transport encryption* is part of the default deployment, whether the solution automatically provides strong default passwords and whether these can be changed in an integrated way, that is, without risking to break the application (*password management*), and whether the solution uses or provides means to verify the trustworthiness of the installation package, for example, by using digital signatures or by providing the source code (*trusted runtime environment*).
4. *Unified interface* shows whether the solution helps manage multiple warehouse instances of different types.
5. *Sustainability* covers 2 subcriteria—*full availability of source code* is important for customizing the solution to local requirements and the *last update* of the installation package is an indicator of whether the solution is actively maintained by the provider of the solution or by the community.

The results of the comparison are presented in Tables 4-5.

As can be seen, our infrastructure is the only off-the-shelf solution supporting both i2b2 and tranSMART. Moreover, our software, the IDRT i2b2 Wizard, and i2b2 Quickstart are the only solutions that fulfill requirement 1 (robust installation of a trusted runtime environment), as the other (cloud-based) solutions are not capable of providing a trusted runtime environment due to the reasons explained above. However, i2b2 Wizard and i2b2 Quickstart are not container-based solutions but rather script-based solutions and thus are significantly less flexible than our tool, which is based on container virtualization. Furthermore, our tool is the only solution that fulfills requirement 2 (unified installation and maintenance) because it provides integrated support for both i2b2 and tranSMART through common commands. Finally, our tool is the only solution that fulfills requirement 3 (built-in security) as it is the only solution that provides out-of-the-box support for multiple important security features, such as transport encryption and strong passwords. The IDRT i2b2 Wizard is quite outdated and has not received updates in more than 2 years.

**Table 4:** Comparison of provisioning infrastructures: Our solution, IDRT^a^ and i2b2^b^ Quickstart.

Feature		Our solution		IDRT^a^ [26]		i2b2^b^ Quickstart [27]	
**Supported target platforms**							
	i2b2 (current major version)		Yes		No		Yes
	tranSMART (current major version)		Yes		No		No
Container based		Yes		No		No	
**Security by default**							
	Transport encryption		Yes		No		No
	Password management		Yes		No		No
	Trusted runtime environment		Yes		Yes		Yes
**Unified interface**							
	Central multi-instance management		Yes		No		No
**Sustainability**							
	Full availability of source code		Yes		Yes		Yes
	Last update		March 2020		August 2017		February 2020

^a^IDRT: Integrated Data Repository Toolkit.

^b^i2b2: Informatics for Integrating Biology and the Bedside.

**Table 5:** Comparison of provisioning infrastructures: i2b2^a^ on Dockerhub, tranSMART on Dockerhub, and manual installation.

Feature		i2b2^a^ on Dockerhub [28]	tranSMART on Dockerhub	Manual installation
**Supported target platforms**				
	i2b2 (current major version)	Yes	No	Yes
	tranSMART (current major version)	No	Yes	Yes
Container based		Yes	Yes	No
**Security by default**				
	Transport encryption	No	No	Yes
	Password management	No	No	Yes
	Trusted runtime environment	No	No	Yes
**Unified interface**				
	Central multi-instance management	No	No	No
**Sustainability**				
	Full availability of source code	No	No	Yes
	Last update	February 2020	October 2019	April 2020

^a^i2b2: Informatics for Integrating Biology and the Bedside.

#### Data-Loading Tools

In addition to transmart-batch and tMDataLoader, which are both used by our solution, there are further data loaders for tranSMART and i2b2. First, transmart-ETL is the standard loading tool for tranSMART. It is included in the standard software installation of tranSMART and is based on the Pentaho Data Integration platform. Second, ICE is a data loading and curation tool supporting a graphical user interface [36]. Third, transmart-copy is a very lightweight loading tool that copies data provided in a tabular form into the tables of the tranSMART database. tmtk is the solution most similar to our approach. It is a Python-based solution that enables the integration of data via a high-level language and several classes. It is typically used in Jupyter notebooks. Analogous to our solution, it uses transmart-batch as a loading tool. It also supports flexible means for organizing data into entities and attributes through an additional graphical tool called the Arborist. Moreover, for i2b2 only, there are other loading tools available. The most comprehensive is the IDRT Import and Mapping Tool [26]. The tool supports various import formats, such as CSV files; provides access to structured query language databases, such as Clinical Data Interchange Standards Consortium (CDISC) Operational Data Model (ODM) [44,45]; and provides direct support for CDMs, that are, for example, used for billing purposes. Talend Open Studio is used for all ETL processes.

We compared these tools with our approach with respect to the following criteria:

1. As in the previous section, the criterion *supported target platforms* shows whether a solution can be used for the current major version of i2b2 (ie, 1.7.x) and/or tranSMART (ie, 16.3).
2. The criterion *EAV schema support* indicates whether the tool supports EAV input data with multiple attribute columns (*multi-column*) or with only one attribute column (*basic*).
3. *Automated data cleansing and preprocessing* covers subcriteria indicating whether the tool can handle *different encodings, data types,* and *syntaxes* for different data sources or if the tool requires all incoming data to conform to a single, predefined specification, and the subsequent subcriteria show whether the tool can handle *missing or invalid data* and *duplicate data* or whether the ETL process is aborted if it encounters one of these anomalies.
4. The criterion *loading strategy* indicates whether the tool employs other data-loading tools (*meta*) or whether the tool implements its own loading procedures (*direct*).
5. *Configuration paradigm* indicates whether the tool configuration follows a declarative approach or an *imperative* approach.
6. The criterion *sustainability*, as in the previous section, covers 2 subcriteria with the same semantics—*full availability of source code* and the *last update*.

The results of the comparison are provided in Tables 6-7.

**Table 6:** Comparison of extraction-transformation-loading tools: Our solution, tranSMART-ETL^a^, tMData-loader, and transmart-batch.

Feature		Our solution		tranSMART-ETL^a^ [33]		tMData-loader [34]		transmart-batch [35]	
**Supported target platforms**									
	i2b2^b^ (current major version)		Yes		No		No		Yes
	tranSMART (current major version)		Yes		Yes		Yes		Yes
EAV^c^ schema support		Multi-column		Basic		Basic		Basic	
**Automated data cleansing and preprocessing**									
	Different encodings, data types, and syntaxes		Yes		No		No		No
	Missing or invalid data		Yes		No		No		No
	Duplicate data		Yes		Yes		No		No
Loading strategy		Meta		Direct		Direct		Direct	
Configuration paradigm		Declarative		Imperative		Imperative		Imperative	
**Sustainability**									
	Source code fully available		Yes		Yes		Yes		Yes
	Last update		March 2020		March 2018		December 2017		June 2016

^a^ETL: extraction-transformation-loading.

^b^i2b2: Informatics for Integrating Biology and the Bedside.

^c^EAV: entity-attribute-value.

**Table 7:** Comparison of extraction-transformation-loading tools: Integrated Curation Environment, Integrated Data Repository Toolkit, transmart-copy, and tmtk^a^.

Feature		ICE^b^ [36]	IDRT^c^ [26]	tranSMART-copy [37]	tmtk^a^ [38]
**Supported target platforms**					
	i2b2^d^ (current major version)	No	No	No	No
	tranSMART (current major version)	Yes	No	Yes	Yes
EAV^e^ schema support		Basic	No	No	Basic
**Automated data cleansing and preprocessing**					
	Different encodings, data types, and syntaxes	No	No	No	No
	Missing or invalid data	No	No	No	Yes
	Duplicate data	No	Yes	No	No
Loading strategy		Meta	Direct	Direct	Meta
Configuration paradigm		Imperative	Imperative	Imperative	Imperative
**Sustainability**					
	Source code fully available	No	Yes	Yes	Yes
	Last update	July 2016	August 2017	December 2019	February 2020

^a^tmtk: TranSMART data curation toolkit.

^b^ICE: Integrated Curation Environment.

^c^IDRT: Integrated Data Repository Toolkit.

^d^i2b2: Informatics for Integrating Biology and the Bedside.

^e^EAV: entity-attribute-value.

As can be seen, our solution and transmart-batch are the only tools to support both i2b2 and tranSMART and thus to fulfill requirement 1 (p*latform independence*). Requirement 2 (*support for different types of data*) is strongly connected to requirement 3 (*automated data cleansing and preprocessing)*. At the structural level, our tool is the only tool to support EAV schema resolution in which multiple columns can be combined (eg, *lab analytes* together with *units of measurement*) and thus is the only one to fulfill requirement 2 (*support for different types of data*). Moreover, our tool is also the only one that is capable of automatically detecting and handling multiple input data properties, such as encodings, syntaxes, and data types, and thus to ingest heterogeneous data often encountered in the clinical context. Our tool and tranSMART‑ETL are both capable of automatically handling duplicate data. In addition to our tool, tmtk and ICE are also meta-loading tools; however, they have fewer data cleansing functionalities. tMDataLoader, ICE, and IDRT are quite outdated and have not received updates in more than 1.5 years.

We conclude that our set of tools is the only solution that supports all requirements outlined in the *Methods* section. Moreover, our solutions are fully open source software, allowing users to maintain their own version if needed, thus decreasing the risks of adoption and improving sustainability.

### Limitations and Future Work

In future work, we plan to address the limitations of the current version of the infrastructure. First, the current implementation does not scale to huge data volumes. At the infrastructure level, this would require support for shared databases. On the data-loading layer, support for processing data in the form of smaller blocks or chunks is needed. Extending the data-loading pipeline with this feature will be relatively straightforward. However, the loading tools used as backends need to support incremental loading, which is currently only supported for i2b2 with the transmart-batch backend. In general, the pipeline would benefit significantly from incremental loading capabilities; therefore, we are exploring options to integrate an incremental loading procedure directly into the software.

An additional area of future improvements is authentication and authorization management. For deployments with a large user base, the use of single sign-on concepts, such as OAuth2 [46], will become relevant. As tranSMART uses Spring Security [47], which supports OAuth2, this should be straightforward to accomplish. However, the software stack used by i2b2 does not support OAuth2 natively. Therefore, we plan to evaluate the approach described by Wagholikar et al [48]. Another limitation in terms of information security is that our use of DCT [31] is currently restricted to checking the authenticity and integrity of the base images when building the images. In future versions, we plan to use DCT to sign images as well, which is particularly important when publishing them on the internet.

The current version of the infrastructure focuses on clinical data or selected genomic variants. TranSMART, however, has built-in support for a wide range of high-dimensional data types (see the *Selection of Target Systems* section). In future work, we plan to add support for loading these types of data as well. Although this will require some effort, such data are typically much more structured and represented in standardized formats than the data considered in this study.

Currently, our loading pipeline focuses on automated structural and syntactic harmonization. Automated mapping procedures to standard terminologies are not yet implemented, mainly because in a first step, we have developed the pipeline following our project-specific requirements. Here, all data sets integrated until now have mostly either been (1) collected in a structured form, using standard terminologies as they were captured; (2) mapped to standard terminologies before they were fed into our pipeline; or (3) loaded for use cases that did not require mapping to semantic standards. However, semantic harmonization is a very important process, and the implementation of interfaces to terminology and ontology services directly into our pipeline is part of our development roadmap.

Finally, we also plan to integrate a wide range of privacy-enhancing technologies into the pipeline. In previous work, we have already integrated a flexible method for data anonymization into an earlier version of our software [49]. Currently, we are working on integrating the pipeline with a HL7 FHIR (Health Level Seven Fast Healthcare Interoperability Resources)–based pseudonymization component.

### Summary and Conclusions

In this paper, we have presented a flexible infrastructure that supports the agile development and provisioning of translational data analytics platforms to researchers. Our solution helps to bridge the interdisciplinary gap between clinicians and informaticians by enabling the creation of data warehousing solutions in an iterative process involving short feedback cycles following a pay-as-you-go approach [15]. We have achieved this by combining a Docker-based (private) cloud infrastructure for managing warehouse instances with a flexible and easy-to-use loading pipeline based on a declarative configuration paradigm. We have used the platform successfully to support a wide range of projects that used different types of data, which we used in our experiments. The solutions described in this paper are available to the community as open source software [19,20].

## Abbreviations

ACHILLES: Automated Characterization of Health Information at Large-scale Longitudinal Evidence Systems
CDISC: Clinical Data Interchange Standards Consortium
CPU: central processing unit
CSV: comma-separated values
CDM: common data model
DCT: Docker Content Trust
DIFUTURE: Data Integration for Future Medicine
EAV: entity-attribute-value
ER: entity-relationship
ETL: extraction-transformation-loading
HL7 FHIR: Health Level Seven Fast Healthcare Interoperability Resources
i2b2: Informatics for Integrating Biology and the Bedside
ICE: Integrated Curation Environment
IDRT: Integrated Data Repository Toolkit
IT: information technology
ODM: Operational Data Model
OHDSI: Observational Health Data Sciences and Informatics
OMOP: Observational Medical Outcomes Partnership
tmtk: TranSMART data curation toolkit
